# Supplementary material for: A Double-Blind, Randomized, Placebo-Controlled Crossover Clinical Study of the Effects of Alpha-s1 Casein Hydrolysate on Sleep Disturbance
Source: Nutrients. 2019 Jun 27;11(7):1466. doi: 10.3390/nu11071466 (PMC6682925; doi:10.3390/nu11071466)
Supplement: Supplementary file 1 [file nutrients-11-01466-s001.pdf]

Supplementary Table 1. The list of dairy products and high-tryptophan concentration foods that were restricted during the study.

Category	List of foods
**Do not eat:**	
Dairy products	Milk, Yogurt, Cheese
Pork Tripe/Chitterlings	Grilled tripe, Tripe hotpot
Beans and related products	Soybean, Tofu, Soy milk, Soy protein powder
Others	Chi-seed, Spirulina
**Limited (≤100g/day):**	
Eggs	2 eggs, 10 quail eggs
Poultry	1 chicken breast, 1 chicken leg, 10 smoked duck slices
Meat	Half portion of pork, 2 piece of tenderloin of lamb/veal
Processed meat	Half of spam, 10 small Vienna Sausages, 10 strips of bacon
Nuts	1 cup of peanut, pumpkin seed, sunflower seed, sesame
Fish	10 slices of smoked salmon, 1 piece of cod, 3/4 can of sea snail

Supplementary Table 2. Differences of change in sleep parameters measured by polysomnography (PSG) between control (Placebo) and test (Alpha-s1 casein hydrolysate (ACH)) phases.

Variables	Placebo		ACH		*P-value*[*]		
	Week 0	Week 4	Week 0	Week 4	Group	Time	Group* Time
Time-in-bed (min)	393.4 ± 4.1	396.1 ± 5.3	393.0 ± 2.7	394.1 ± 4.4	0.718	0.613	0.841
Total sleep time (min)	326.5 ± 8.9	332.1 ± 8.1	330.6 ± 8.9	341.5 ± 9.0	0.352	0.286	0.729
Sleep latency (min)	8.7 ± 1.7	8.1 ± 1.4	10.7 ± 2.5	6.5 ± 0.9	0.808	0.620	0.437
Sleep efficiency (%)	83.0 ± 2.1	83.8 ± 1.7	84.0 ± 2.1	86.6 ± 1.9	0.183	0.324	0.599
WASO (min)	58.2 ± 8.1	55.9 ± 5.9	51.8 ± 7.6	46.1 ± 7.3	0.040	0.567	0.551
WASO (%)	15.1 ± 2.1	14.5 ± 1.6	13.6 ± 2.1	12.0 ± 1.9	0.055	0.530	0.545
Total arousal (N)	78.7 ± 6.4	80.9 ± 6.6	73.6 ± 6.4	77.9 ± 7.2	0.352	0.453	0.812
Arousal index (/hr)	15.0 ± 1.4	14.8 ± 1.2	13.7 ± 1.3	14.3 ± 1.5	0.196	0.841	0.665
NREM/TST (%)	82.3 ± 1.2	80.5 ± 1.2	80.3 ± 1.2	78.0 ± 1.2	0.056	0.291	0.483
Stage N1/TST (%)	16.9 ± 1.8	16.1 ± 1.2	15.9 ± 1.6	15.9 ± 1.6	0.366	0.826	0.917
Stage N2/TST (%)	59.1 ± 2.1	57.8 ± 2.0	56.4 ± 2.9	60.9 ± 1.4	0.965	0.385	0.116
SWS/TST (%)	6.3 ± 1.4	6.6 ± 1.4	5.4 ± 1.3	3.2 ± 0.9	0.046	0.258	0.156
REM/TST (%)	17.7 ± 1.2	19.5 ± 1.2	19.8 ± 1.2	20.0 ± 1.2	0.042	0.314	0.440
REM latency (min)	105.2 ± 13.5	111.1 ± 13.9	105.1 ± 12.9	108.7 ± 13.3	0.973	0.429	0.698
AHI (/hr)	4.1 ± 1.7	3.8 ± 1.7	4.5 ± 1. 7	3.8 ± 1.7	0.574	0.299	0.661

PLM index (/hr)	7.6 ± 3.9	7.4 ± 3.9	8.0 ± 3.9	7.2 ± 3.9	0.915	0.501	0.749

Data are mean ± SE (standard error).

*Linear mixed-effect model was used to analyze the effects of group, time and group*time for 4 weeks.

WASO = wake after sleep onset, TST = total sleep time, NREM = non-rapid eye movement, SWS = slow wave sleep, REM = rapid eye movement, AHI = apnea-hypopnea index, PLM = periodic leg movement

Supplementary Table 3. Analysis of vital signs and laboratory examinations at baseline (week 0) and after 4 weeks of placebo or ACH administration (week 4).

Variables		Placebo	ACH	*P-value*[*]
Systolic blood pressure (mmHg)	Week 0	113.9 ± 2.2	116.1 ± 2.1	0.453
	Week 4	112.2 ± 2.2	113.3 ± 2.2	0.723
Diastolic blood pressure (mmHg)	Week 0	72.8 ± 1.6	71.5 ± 1.7	0.574
	Week 4	73.0 ± 1.8	72.5 ± 1.5	0.838
Pulse rate (beats/min)	Week 0	72.7 ± 1.6	69.2 ± 1.4	0.108
	Week 4	70.1 ± 1.3	71.7 ± 1.3	0.348
Body temperature (℃)	Week 0	36.5 ± 0.0	36.4 ± 0.0	0.558
	Week 4	36.5 ± 0.0	36.5 ± 0.1	0.425
White blood cell (10^3/μL)	Week 0	5.7 ± 0.3	5.6 ± 0.2	0.866
	Week 4	5.7 ± 0.3	5.6 ± 0.3	0.745
Red blood cell (10^6/μL)	Week 0	4.5 ± 0.1	4.5 ± 0.1	0.900
	Week 4	4.5 ± 0.1	4.4 ± 0.1	0.623
Hemoglobin (g/dL)	Week 0	13.4 ± 0.2	13.5 ± 0.2	0.743
	Week 4	13.4 ± 0.2	13.4 ± 0.2	0.881
Platelet (10^3/μL)	Week 0	255.4 ± 8.2	257.8 ± 8.0	0.841
	Week 4	251.9 ± 8.2	258.8 ± 8.7	0.566
Lymphocyte (%)	Week 0	37.3 ± 1.3	38.9 ± 1.1	0.337
	Week 4	38.5 ± 1.2	38.1 ± 1.2	0.805
Monocyte (%)	Week 0	7.4 ± 0.3	6.8 ± 0.3	0.188
	Week 4	7.2 ± 0.3	7.2 ± 0.3	0.976
AST (IU/L)	Week 0	26.3 ± 2.3	23.1 ± 0.8	0.261
	Week 4	23.0 ± 0.9	23.8 ± 1.2	0.654
ALT (IU/L)	Week 0	24.3 ± 3.0	21.2 ± 1.7	0.467
	Week 4	20.5 ± 1.7	19.9 ± 1.1	0.883
Triglyceride (mg/dL)	Week 0	100.5 ± 14.1	102.9 ± 11.8	0.893
	Week 4	96.7 ± 10.3	94.5 ± 8.9	0.871
Total cholesterol (mg/dL)	Week 0	182.9 ± 5.1	181.6 ± 4.1	0.852
	Week 4	183.7 ± 4.6	183.2 ± 5.3	0.946
LDL-cholesterol (mg/dL)	Week 0	113.0 ± 4.8	110.0 ± 3.8	0.632
	Week 4	111.1 ± 4.4	111.6 ± 4.6	0.940

Data are mean ± SE (standard error).

Student's t-test was used to compare the difference between the groups.
